# Update on Locoregional Therapies for Liver Cancer: Radiation Segmentectomy

**DOI:** 10.3390/curroncol30120732

**Published:** 2023-11-23

**Authors:** Farnaz Dadrass, Alex Sher, Edward Kim

**Affiliations:** Diagnostic, Molecular and Interventional Radiology, Mount Sinai Hospital, 1468 Madison Ave, New York, NY 10029, USA; alex.sher2@mountsinai.org (A.S.); edward.kim@mountsinai.org (E.K.)

**Keywords:** interventional oncology, radiation segmentectomy, Y90 radioembolization

## Abstract

Over 900,000 people worldwide were diagnosed with liver cancer in 2022 alone, with hepatocellular carcinoma (HCC) accounting for 75–85% of cases. Treatment for HCC includes some combination of systemic therapies, surgery, liver transplantation, ablation, and intra-arterial therapies with transarterial chemoembolization (TACE) or transarterial radioembolization (TARE). Currently, the Barcelona Clinic Liver Cancer (BCLC) guidelines have acknowledged liver transplantation, surgical resection, and thermal ablation as curative therapies in very early to early stage HCC (BCLC-0 and BCLC-A). While these modalities are the preferred curative treatments for a very early to early stage disease, there are challenges associated with these options, such as organ availability and patient eligibility. Current data shows the role of radiation segmentectomy as a curative therapeutic option for very early to early stage HCC that is unresectable and not amenable to ablation. As future data continues to elucidate the ability for radiation segmentectomy to achieve complete pathologic necrosis, the goal is for the BCLC staging model to acknowledge its role as a curative treatment in this patient population and incorporate it into the ever-evolving guidelines.

## 1. Introduction

Over 900,000 people worldwide were diagnosed with liver cancer in 2022 alone, with hepatocellular carcinoma (HCC) accounting for 75–85% of cases [1,2]. The main risk factors for HCC include the following: chronic infection with hepatitis B virus (HBV) or hepatitis C virus (HCV), aflatoxin-contaminated foods, alcohol use disorder, obesity, type 2 diabetes mellitus, and smoking [1,3]. While the rate of viral hepatitis-related malignancy has been decreasing worldwide over the past few decades—largely due to the invention of vaccines and anti-viral treatments –the rate of non-alcoholic fatty liver disease (NAFLD) and non-alcoholic steatohepatitis (NASH)-related malignancy has increased [1]. 

The diagnosis of HCC can be made with imaging or with tissue biopsy in patients with cirrhosis. Using CT or MRI, the liver imaging reporting and data system (LI-RADS) criteria can diagnose patients without the need for tissue sampling [4]. This is important as liver dysfunction can result in a high risk for bleeding. Diagnosis of HCC via imaging is performed with multiple phases. The tumor will demonstrate enhancement in the early arterial phase, meaning it will look bright after the administration of an intravenous contrast when compared to the surrounding liver parenchyma. This is followed by temporal decreased enhancement, which becomes darker than the adjacent liver parenchyma, and is referred to as a “delayed venous washout”. This results in a peripheral rim, or “pseudocapsule”, which is highly specific for HCC [4]. The prognosis for HCC is dependent on patient and tumor characteristics, such as tumor burden, extrahepatic spread, vascular infiltration, and tumor differentiation. Notably, HCC is also heavily influenced by underlying liver disease and liver function. Elevated AFP levels are significantly associated with mortality and have been shown to predict the risk of tumor recurrence after a resection and liver transplantation [2].

The Barcelona Clinic Liver Cancer (BCLC) system is a widely recognized framework for staging, prognosis, and treatment guidance for HCC. This comprehensive system considers various factors, including tumor characteristics (such as size, number, vascular invasion, and extrahepatic disease), performative status (measured with the Eastern Cooperative Oncology Group or ECOG scale), and liver function (evaluated using the Child–Pugh score calculated by using the lab values for bilirubin, albumin, INR, encephalopathy, and the presence of ascites) [5]. BCLC categorizes HCC into distinct stages, each associated with specific criteria, and provides corresponding treatment recommendations. These stages include Very Early Stage (BCLC 0), Early Stage (BCLC-A), Intermediate Stage (BCLD-B), Advanced Stage (BCLC-C), and End Stage (BCLC-D), each associated with their respective recommendations [6]. 

The management of HCC consists of a range of therapeutic modalities, including, but not limited to, surgery, liver transplantation, ablation, and intra-arterial therapies, such as transarterial chemoembolization (TACE) and transarterial radioembolization (TARE). Over the past two decades, the rates of treatment with locoregional therapy (LRT) have grown significantly, with 42.3% of patients awaiting liver transplantation in 2003 receiving some form of LRT—now up to 92.4% in 2018 [7]. Notably, there was a substantial increase in treatment with radioembolization, with less than 5% LRT consisting of radioembolization in 2013—up to 19% in 2018 [7]. In this review article, we will discuss the current BCLC guidelines, with a focus on very early to early stage HCC, and explore the literature that discusses the evolving role of radiation segmentectomy in this patient population. 

## 2. Navigating Very Early to Early Stage (BCLC 0-A) HCC

The BCLC (consisting of guidelines), introduced in 1999 and updated throughout the years, serves as the most commonly used staging system for HCC in Western countries [8,9,10,11,12,13,14]. 

### 2.1. BCLC-0

Very early stage HCC (BCLC-0) is defined as the presence of a solitary lesion measuring 2 cm of less, without evidence of vascular invasion or extrahepatic spread. Patients falling into this category must demonstrate preserved liver function and remain free of cancer-related symptoms [6]. Surgical resection, with the ultimate goal of liver transplantation, is the first line of therapy due to the high risk of recurrence. However, there are various factors to consider, including local regulations governing enlistment and priority policies pertaining to patients who are also surgical candidates. 

In cases where liver resection or transplantation are not viable options for curative intent, the recommended initial treatment, as outlined by the guidelines, would be thermal ablation. Recently, the BCLC guidelines have incorporated both TACE and TARE for treatment failures or patients deemed to be poor ablation candidates. For individuals in this stage with severe liver dysfunction and decompensation, liver transplantation remains the ideal option, as they receive higher priority due to their end-stage liver status.

### 2.2. BCLC-A

Early stage HCC (BCLC-A) is defined as solitary HCC, irrespective of size, or 3 lesions up to 3 cm. Required criteria within this category include the absence of macrovascular invasion, extrahepatic spread, and cancer-related symptoms, while maintaining preserved liver function. 

For solitary BCLC-A HCC, therapeutic planning is approached in the context of whether the patient has clinically significant portal hypertension (CSPH). CSPH is defined as a hepatic venous gradient pressure of greater than 10 mmHg and is associated with higher rates of postoperative complications [6,8]. Surrogates of CSPH include ascites, splenomegaly, varices, and thrombocytopenia. For patients who do not have CSPH, surgical resection is typically the first line treatment option. This is largely due to the ability to assess the pathologic profile and biomarkers of the resected liver to determine the risk of recurrence. For those who are high risk, liver transplantation should be considered. In the absence of such markers, the less invasive and less costly option, thermal ablation, is recommended for lesions ≤ 3 cm that are not in high-risk locations. 

Multifocal BCLC-A HCC recommendations favor liver transplantation due to the high risk of recurrence [6]. If liver transplantation is not feasible, the recommendations do not specify between surgical resection and ablation. 

These guidelines are further summarized in Table 1. 

This chart provides a simplified version of the recommendations, as detailed in the section above. For patients with BCLC-0 HCC and CSPH, liver transplantation (LT) can be considered for patients with acceptable tumor burden; otherwise, the recommendation is to proceed with thermal ablation. For a select group of patients with solitary, peripheral HCC and an adequate liver remnant, surgical resection (SR) can be considered. 

## 3. Locoregional Therapies Available for BCLC 0-A

### 3.1. Thermal Ablation

Thermal ablation, either performed with radiofrequencies or microwaves, has demonstrated a curative capacity for HCC lesions measuring up to 3 cm, and has such been incorporated into the treatment algorithm for patients with BCLC 0 or A HCC [15]. The goal of this therapy is to achieve complete thermal coagulation necrosis of the tumor, ideally leaving no viable malignant tissue behind with adequate margins. Nonetheless, there are geographic limitations that may result in incomplete tumor necrosis and higher rates of recurrences, or even complications [15]. These locations mainly include the hepatic dome, caudate lobe, major intrahepatic vessels, bowel, gallbladder, and colon [15]. For lesions greater than 3 cm, there are additional limitations associated with ablation zone size and overlap, which puts patients at an increased risk for local recurrence [16,17,18]. The local recurrence rate thus increases due to insufficient ablation margins. It has been established that local tumor size is an independent prognostic factor for long-term survival following ablation. 

### 3.2. Transarterial Chemoembolization

Transarterial chemoembolization (TACE) is a standard locoregional therapy, more frequently used in the treatment of intermediate-stage HCC. There are two main types of TACE, conventional TACE (cTACE) or drug-eluting beads TACE (DEB-TACE). When performing cTACE, an intraarterial injection of cytotoxic agents (such as doxorubicin or cisplatin) is emulsified in an oil-based radiopaque agent (typically lipiodol), followed by the injection of an embolic agent. DEB-TACE contains non-absorbable embolic microspheres that release drugs with simultaneous cytotoxic and tumor embolic effects. TACE failure or refractoriness has been repeatedly described in the literature, defined as insufficient necrosis of intrahepatic lesions, emergence of new lesions within three months, or local recurrence within three months post-TACE [19,20]. This led to the combination treatment of TACE with ablation for solitary recurrent HCC < 5 cm, which shows improved overall survival when compared to ablation alone [21]. While this has been a more effective treatment option, repeated treatments with TACE can lead to decreased liver function [19]. 

### 3.3. Transarterial Radioembolization

Radioembolization performed with yittrium-90, also commonly referred to as selective internal radiation therapy (SIRT) or trans arterial radioembolization (TARE), or more colloquially as Y90, uses internal radiation therapy to target and destroy tumors. The procedure involves selective administration of an intraarterial ablative dose of Y90 microspheres. Given the increased arterial supply of the tumor(s), as well as the increased tumor to normal flow ratio, most of the treatment is delivered preferentially to the tumor compared to the background liver. Furthermore, by performing a selective injection, most of the non-involved hepatic parenchyma is spared, allowing for high doses of radiation to be safely delivered. 

When delivered to two or less Couinaud segments, the technique is called radiation segmentectomy, with the concept similar to that of anatomic surgical resection. Some technical advantages of radiation segmentectomy over thermal ablative techniques include a decreased bleeding risk and a decreased risk of tract seeding, which can be associated with a transhepatic approach as opposed to a transarterial approach [22]. Additionally, it decreases the risks associated with transhepatic thermal ablation, such as potential damage to surrounding structures or incomplete treatment in lesions near major vascular and biliary structures, hallow viscera, and the diaphragm [7,22].

Currently, there are two FDA-approved Y90 microsphere products available, with only glass microspheres FDA-approved for the treatment of HCC [23]. The glass spheres have a size of 25 ± 10 μm and resin spheres have a size of 32 ± 10 μm, allowing for distribution preferentially within the tumor [24]. The starting calibration doses range from 3 to 20 GBq vials for glass microspheres with corresponding 1.2–20 million microspheres. For resin, the starting dose is 10 GBq, which contains approximately 50 million microspheres. The activity in glass is 2500 Bq versus 50 Bq in resin. Therefore, while there are lower numbers of microspheres in the standard dose in glass microspheres, the activity is significantly higher. As a result, the distribution pattern may vary as the cluster size differs with the number of microspheres. Notably, only glass microspheres were used in the studies described in this review. 

### 3.4. Radiation Segmentectomy Current Guidelines

While surgical resection and ablation are considered curative treatment options for BCLC 0-A lesions that meet specified criteria, radiation segmentectomy has emerged as a viable alternative for single nodules ≤ 8 cm that are not suitable for surgical resection or ablation. The guidelines also acknowledge the role of radiation segmentectomy to increase remnant liver volume in patients with BCLC-A HCC, thereby aiding patients considered for surgical resection [6]. In cases where patients are awaiting liver transplantation with an anticipated waiting period exceeding 6 months, either TACE or TARE may be used to prevent treatment progression and observe tumor biology. For patients with BCLC-A HCC who are ineligible for ablation, surgical resection, or liver transplantation, and who meet ‘The LEGACY Study’ inclusion criteria (discussed later), the option of combined treatment with TARE alongside TACE can be considered.

While the inclusion of TARE in the 2022 update of the BCLC guidelines marks a significant milestone, ongoing research consistently underscores the effectiveness of radiation segmentectomy as a curative treatment option, placing it on par with the existing curative therapeutic options [25,26,27].

Though not the focus of this review, it is important to note that radioembolization has different treatment goals in subsequent BCLC staging. In intermediate-stage (BCLC-B) HCC, both TACE and TARE can be used downstage by reducing tumor burden for the remaining tumor to fall within the acceptable criteria for liver transplantation. Consequently, in a recent study published by Dhondt et al. in 2022, TARE conferred superior tumor control when compared with chemoembolization in early or intermediate HCC [28].

## 4. Background on Radiation Segmentectomy

One of the first trials that evaluated the efficacy of radiation segmentectomy was a multicenter analysis conducted by Vouche et al. in 2014 [29]. This comprehensive study focused on patients who had undergone radiation segmentectomy with previously untreated, unresectable, solitary HCC measuring 5 cm or less, and which were not amenable to resection or ablation [29]. Administered dose, response rate, time to progression (using the modified Response Evaluation Criteria in Solid Tumors [mRECIST]), radiology–pathology correlation, and long-term survival were assessed. Among the 102 patients included in the study, a complete response was observed in 47%, a partial response in 39%, and a stable disease in 12%. Pathologic examination revealed 100% necrosis in 52% of patients, while 48% exhibited necrosis ranging from 50 to 99%. These findings highlighted a strong correlation between the radiological response and pathologic necrosis, indicating effective treatment and destruction of targeted tumor cells. Notably, an increased survival benefit and better local tumor control were seen with a dose of >190–200 Gy.

Lewandowski et al. investigated the role of radiation segmentectomy as a potential curative treatment in 2018 [25]. Similarly, this retrospective study evaluated patients who had undergone radiation segmentectomy with solitary HCC measuring 5 cm or less, which were not amenable to surgical resection or tumor ablation. Building upon insights noted by Vouche et al., the authors selected patients who had received a dose exceeding 190 Gy [25]. Their findings indicated that 90% of patients exhibited a response based on the European Association for the Study of the Liver (EASL) criteria, with 59% of them showing a complete response. When evaluated using the World Health Organization (WHO) criteria, 71% of patients showed a response, including 16% with a complete response. This study also indicated a local tumor control rate of 72% over a 5-year period, with a mean time to progression of 2.4 years. Overall survival at 5 years was 55%, with a 5-year survival of 75% for patients with HCC measuring ≤ 3 cm. These results mirror those seen with other curative treatment options for early stage HCC. This indicated that radiation segmentectomy could indeed serve as therapy with a curative intent for BCLC 0-A HCC in patients with solitary lesions measuring 5 cm or less, which were not amenable to ablation or resection. This study further demonstrated prolonged survival, similar to that of surgical resection and thermal ablation, with a favorable adverse event profile.

‘The LEGACY Study’, conducted by Salem et al., played a pivotal role in establishing the effectiveness of radioembolization using Y90 glass-based microspheres to treat solitary, unresectable HCC [26]. This multicenter, single-arm, retrospective study evaluated the objective response rates and duration of responses in 162 patients with solitary HCC measuring ≤ 8 cm, Child–Pugh A cirrhosis, and ECOG performance status 0–1. Of these patients, 21% received radioembolization as neoadjuvant therapy prior to transplantation, 6.8% prior to resection, and all others were intended as the primary treatment. Within this patient cohort, 60.5% were staged as BCLC-A and 39.5% were staged as BCLC-C. The median tumor size was 2.7 cm, with a range of 1.0–8.1. The majority of patients received selective infusions (95.7%), and 1.9% received lobar infusions, with 2.5% of patients receiving mixed infusions. The best objective response rate was 88.3%. Additionally, 76.1% of participants maintained their responses for a duration of 6 months or longer. Notably, the local tumor recurrence rates observed in this cohort were competitive with those of thermal ablation [26]. 

The prolonged patient benefit and durable responses in ‘The LEGACY Study’ further elucidated the role of radiation segmentectomy as a curative therapy in the setting of BCLC-A HCC. It also showed the role of neoadjuvant treatment for downstaging and bridging to liver transplantation. Moreover, this study provided a new potential threshold dose for achieving an ablative effect, as patients consistently exhibited CPN when their absorbed dose surpassed 400 Gy within the perfused volume. Even at this, dose treatment was found to be safe as only 31 out of 162 patients had grade 3 adverse events, as well as only 1 grade 4 and grade 5 event each, most of which resolved during the time of the study period [26].

Due to the results seen with ‘The LEGACY Study’ trial, TARE has received FDA approval for the treatment of solitary HCC, and it has since been incorporated into the BCLC treatment algorithm for patients classified as BCLC stage 0-A. 

The prospective phase 2 study, known as ‘Radiation segmentectomy for curative intent of unresectable very early to early stage hepatocellular carcinoma (RASER)’, cemented the role of radiation segmentectomy curative-intent treatment for solitary very early to early stage HCC and deemed it surgically unresectable and unsuitable for ablation [27]. This study marked the first prospective evaluation of its kind, specifically focusing on individuals with unresectable, solitary early to early stage HCC [27]. A sustained complete response was experienced by 90% of patients after a single treatment, with a median duration of response of 635 days. The majority of adverse events reported were grade 1–2 Clavien Dindo, reaffirming the safety profile of radiation segmentectomy, despite the delivery of high radiation doses exceeding 1000 Gy to the tumor, as measured using post Y90 PET/CT. This prospective study provides evidence that radiation segmentectomy can yield a sustained complete response with a low incidence of high-grade adverse effects in unresectable very early to early stage HCC, particularly for those for whom ablation is not a viable option. These results align closely with the outcomes observed in other retrospective studies exploring the use of radioembolization in early stage HCC [25,26,29]. This opens for discussion the role of radiation segmentectomy as a curative intent treatment, in lieu of ablation or resection.

## 5. Complete Pathologic Necrosis

When discussing the role of radiation segmentectomy as a curative treatment option, the achievement of complete pathologic necrosis (CPN) is an important consideration. The study conducted by Vouche et al., as described above, initially demonstrated an association between CPN in HCC with a radioembolization radiation dose of > 190 Gy in 2014 [29]. Subsequent studies have since evaluated the correlation between an increased absorbed dose and CPN.

In 2021, a subset analysis of ‘The LEGACY Study’ data published by Gabr et al. evaluated patients who underwent Y90 glass microsphere radiation segmentectomy from 2014 to 2017 and later received liver transplantation or resection. Their explanted livers were evaluated for CPN [30]. This study included patients with treatment-naïve, solitary HCC ≤ 8 cm, with preserved liver function (Child–Pugh A). Of the participants, 76% underwent liver transplantation and 24% underwent resection. The median dose was 240 Gy, with 67% CPN, 22% extensive necrosis, and 5% partial necrosis at explant. A total of 86% of patients with a dose > 190 Gy achieved CPN, while 65% of patients who received < 190 Gy did not. All patients who received > 400 Gy exhibited CPN. The median tumor size was 2.5 cm. While the absorbed radiation dose was significantly associated with CPN, the size of the lesion was not. This study helped to validate the relationship between the absorbed radiation dose and the extent of tissue necrosis, finding that a dose of greater than 400 Gy for radiation segmentectomy may be a potential threshold to achieve consistent CPN. 

In 2022, Montezeri et al. was the study is the largest cumulative radiopathologic analysis to date in patients treated with radiation segmentectomy prior to liver transplantation [31]. It evaluated whether Y90 glass microsphere radiation segmentectomy intensification correlated with an increased rate of complete pathologic necrosis (CPN) through explant analysis. The dosage was calculated using the Medical Internal Radiation Dose (MIRD) single-compartment methodology, with a dose of ≥400 Gy and a specific activity of ≥297 Bq administered in the intensification group whenever it was feasible. The dose and specific activity were both significantly higher in the treatment intensification cohort with a median dose of 536 Gy compared to 314 Gy in the baseline cohort, and specific activity of 715 Bq compared to 321 Bq. 

Histopathologic analysis showed CPN in 76% of the treatment intensification cohort compared to 49% in the baseline cohort. Significant differences were seen between the dose, specific activity, and total treatment activity in those with CPN versus without. This study helped to push the boundaries for the appropriate dose for administration in radiation segmentectomy, showing that a dose of ≥446 Gy and a specific gravity of ≥327 Bq were more likely to achieve CPN. It is important to note that there was a stronger association between increased specific gravity and CPN than the increased dose and CPN. Nevertheless, the rate of CPN in 76% of patients in the intensification cohort further confirmed the ablative capacity of radiation segmentectomy.

In 2020, DiNorcia et al. evaluated whether the extent of a tumor response to locoregional therapy could predict the success of liver transplantation and patient outcomes after the procedure [32]. The findings demonstrated that the degree of pathologic responses to locoregional therapy was strongly predictive of positive patient outcomes after liver transplantation for HCC complete pathologic responses, and was significantly associated with lower post-transplant recurrence and superior survival. 

**Figure 1:**
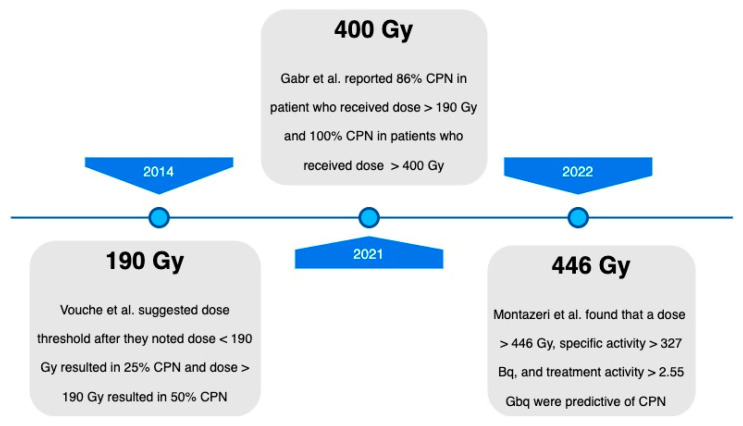
Complete pathologic necrosis threshold dose timeline [29,30,31].

Ultimately, the objective of locoregional therapies should center on achieving CPN, and the recent literature on radiation segmentectomy has consistently demonstrated a curative potential across multiple prospective and retrospective studies. Additionally, it has emphasized its role in the context of downstaging and bridging therapy to liver transplantation. It is noteworthy that radiation segmentectomy has not only proven to be more effective but also maintains an ample safety profile as the dose threshold has increased over the years, as highlighted in Figure 1. Moving forward, it is imperative to refine the treatment parameters to optimize tumor responses and consequently minimize the number of required treatments needed to achieve CPN, and therefore a curative response.

Prominent studies investigating the dosage threshold required to achieve complete pathologic necrosis (CPN) following radiation segmentectomy.

## 6. Non-HCC Lesions

At present, the standard of care for managing metastatic liver malignancies originating from colorectal cancer, neuroendocrine tumors, sarcoma, and various other sources, and primarily revolves around surgical resection, provided the patients meet the eligibility criteria [33]. However, in instances where surgical resection is not feasible due to factors such as anatomic location, medical comorbidities, or the inability to interrupt ongoing chemotherapy for the necessary duration, thermal ablation emerges as a viable alternative. However, this is only the case for lesions measuring 3 cm or less, which have well-defined margins, and are not near any heat-sensitive structures, similar to HCC [34]. 

Given the promising outcomes seen with radiation segmentectomy in the context of HCC, Padia et al. conducted a study in 2021 titled, ‘Yttrium-90 radiation segmentectomy for hepatic metastases: A multi-institutional study of safety and efficacy.’ The authors aimed to assess the potential curative role of radiation segmentectomy in metastatic liver disease [33]. This study included patients from two centers who had undergone Y90 radiation segmentectomy with glass microspheres between 2013 and 2018. However, unlike previous research focused on HCC, this study centered on hepatic metastases. These metastatic lesions were not amenable to surgical resection or ablation and exhibited no signs of progressive extrahepatic disease. Patients also had an ECOG performance status of 0–1 and maintained normal baseline liver function. The median tumor size was 3.6 cm. The tumor response was assessed using the Response Evaluation Criteria in Solid Tumors (RECIST) criteria, with an additional imaging evaluation using mRECIST for hypervascular tumors. The results revealed a tumor RECIST control rate of 92%, encompassing 28% partial responses and 64% of cases with a stable disease. Hypervascular tumors, when evaluated using mRECIST, exhibited an objective response rate of 100%. Furthermore, this study reported an overall survival of 96% at 6 months and 83% at 12 months [33]. These favorable findings contribute significantly to the growing body of evidence supporting the role of radiation segmentectomy as a viable therapeutic option for hepatic metastases, particularly when surgical resection or ablation is not feasible. However, further research is needed to determine whether curative treatment can be consistently achieved in this patient population.

## 7. Conclusions

Hepatocellular carcinoma (HCC) is a major cause of morbidity and mortality [1]. Over the recent decade, the landscape for the management of HCC has significantly evolved, with several modalities available to treat various stages of this disease. Traditionally, liver transplantation and surgical resection have been the mainstay for curative treatments, with 5-year survival rates ranging from 60 to 80% in patients with BCLC-0 and BCLC-A HCC [13]. However, liver transplantation faces limitations due to organ scarcity, resulting in prolonged wait times and even disease progression in some cases. Surgical resection, when feasible, is contingent on strict eligibility criteria and the absence of complicating medical comorbidities. Curative treatment options have been broadened to include thermal ablation for qualifying patients, but remain limited by lesion size and number, as well as geographical constraints [15]. 

Radiation segmentectomy has emerged as a promising approach for achieving complete pathologic necrosis (CPN) in BCLC 0-A HCC, making it a potential curative treatment for patients who are not suitable candidates for surgery or ablation. Research has revealed that higher radiation doses correlate with improved CPN rates, and refinements in treatment parameters have further enhanced tumor response optimizations [29,30,31,32]. Moreover, the use of radiation segmentectomy has shown promise across various BCLC stages and beyond HCC, demonstrating efficacy in the treatment of unresectable hepatic metastases originating from other malignancies. This broadens its potential applications, particularly when surgical resection or ablation is not feasible. As the current literature continues to expand, the goal is for the BCLC staging model to acknowledge its role as a curative treatment in this patient population and for it to be incorporated into the ever-evolving guidelines. 

## Figures and Tables

**Table 1:** Summary of BCLC 0-A recommended therapies.

BCLC Stage	Description	Curative Therapies	Considerations	Additional Therapies
Very early (BCLC-0)	Solitary HCC ≤ 2 cm Preserved liver function	Liver transplantation Surgical resection Thermal Ablation	Liver transplantation candidacy: Not LT candidate → ablation LT candidate with no CSPH → SR LT candidate with CSPH → Ablation or LT	TARE (solitary lesion ≤ 8 cm) TACE
Early (BCLC-A)	Solitary HCC any size or HCC with ≤3 lesions all ≤3 cm Preserved liver function	Liver transplantation Surgical resection Thermal Ablation (for lesions ≤ 3 cm)	CSPH: Absent → SR Present and LT candidate → LT Present and not LT candidate → ablation	TARE (solitary lesion ≤ 8 cm) TACE
